# Case report: Suspected transfusion-related acute lung injury type II in a child with refractory systemic juvenile idiopathic arthritis complicated by macrophage activation syndrome

**DOI:** 10.3389/fped.2023.1237111

**Published:** 2024-01-08

**Authors:** Alenka Gagro, Maja Tomičić, Ivančica Škarić, Barbara Dawidowsky

**Affiliations:** ^1^ Department of Pediatrics, Zagreb Children’s Hospital, School of Medicine, University of Zagreb, Zagreb, Croatia; ^2^ School of Medicine, Josip Juraj Strossmayer University of Osijek, Osijek, Croatia; ^3^ Department of Platelet and Leukocyte Diagnosis and Hemostasis, Croatian Institute of Transfusion Medicine, Zagreb, Croatia; ^4^ Department of Anesthesiology, Resuscitation and Intensive Care Medicine, Zagreb Children’s Hospital, Zagreb, Croatia; ^5^ Department of Pediatric Surgery, Zagreb Children’s Hospital, Zagreb, Croatia

**Keywords:** transfusion-related acute lung injury (TRALI), systemic juvenile idiopathic arthritis (sJIA), macrophage activation syndrome (MAS), cytopenia, thrombocytopenia, platelets

## Abstract

**Introduction:**

Transfusion-related acute lung injury is a rare but potentially fatal complication, which may appear during or post-transfusion of blood products. Patients with macrophage activation syndrome, a serious life-threatening complication associated with systemic juvenile idiopathic arthritis, often require transfusion or administration of blood products for correction of cytopenia, coagulopathy and hypofibrinogenemia.

**Case report:**

A 6-year-old girl with a past medical history of systemic juvenile idiopathic arthritis had the first relapse of the disease during which she developed macrophage activation syndrome. During this life-threatening complication, she received a second dose of whole blood derived filtered and irradiated platelets from a single male donor due to profound thrombocytopenia. Approximately one hour post-infusion, the patient developed progressive dyspnea, hypoxemia and bilateral pulmonary edema. She was promptly intubated and placed on mechanical ventilation for 40 h. Clinical, laboratory and radiological findings, as well as the success of supportive ventilation therapy were highly suggestive of transfusion-related acute lung injury, a life-threatening complication that occurs within six hours of blood component transfusion. Blood immunology showed no presence of anti-human neutrophil antigen and anti-leukocyte antigen class I and class II antibodies in the donor's or patient's plasma.

**Conclusion:**

To the best of our knowledge, we report the first case of a child with systemic juvenile idiopathic arthritis complicated with macrophage activation syndrome who developed type II transfusion-related acute lung injury following platelet transfusion. It is important to consider transfusion-related acute lung injury in transfusion settings in these children and apply critical and restrictive approach for platelet transfusion.

## 1 Introduction

Systemic juvenile idiopathic arthritis (sJIA) is a rare type of juvenile idiopathic arthritis (JIA) characterized by high fever and extra-articular findings, triggered by presumed dysregulation of the innate immune system (1–3). In contrast to other types of JIA, sJIA has a disproportionally higher rate of complications, especially macrophage activation syndrome (MAS) that requires early recognition and prompt therapy (4–7). MAS can result in progressive multi-organ failure and eventually a fatal outcome in 4.1%–15.2% of affected children, making timely diagnosis and prompt initiation of appropriate treatment imperative (5). Based on the severity of macrophage activation syndrome that often leads to profound anemia, thrombocytopenia, hypofibrinogenemia and coagulation disorder triggered by hyperinflammation, some children will require concentrated red blood cells, platelet pools, intravenous fibrinogen and fresh frozen plasma. However, the potential risk of adverse events, including transfusion-related acute lung injury (TRALI) following transfusion of blood or blood products in these patients has not been investigated in detail to date.

TRALI is defined by the presence of respiratory insufficiency and hypoxemia that develop during or within six hours of transfusion of blood or blood products, and imaging will reveal bilateral fluffy infiltrates consistent with pulmonary edema. Although TRALI is considered a rare complication, it is the leading cause of transfusion related-mortality (8–10). Based on a recent consensus redefinition of TRALI, the terminology of TRALI type I [without acute respiratory distress syndrome (ARDS) risk factor] and TRALI type II (with ARDS risk factor or with mild existing ARDS) has been proposed (9). Despite mitigation strategies that include exclusion of females from plasma donation or exclusion of females with a history of pregnancy or known anti-leukocyte antibody, as well as the recent update of TRALI definition, this life-threatening condition often remains unrecognized and under-reported (10).

Clinical data on the pediatric population showed that TRALI is relatively common in critically ill children and indicate several independent risk factors for TRALI, such as high Pediatric Risk of Mortality III score on admission, mechanical ventilation, and sepsis (11). Blood derivative transfusion-related risk factors in children with sJIA complicated by MAS are not reported in detail to date. There are no specific transfusion threshold guidelines in patients with this complication either. A long-term extension study of phase III pivotal trials of canakinumab treatment in patients with sJIA and active systemic features recorded one patient diagnosed with TRALI but no additional data on the possible trigger or type of TRALI were provided (12).

We describe an unusual case of a girl previously diagnosed with sJIA complicated by MAS who received whole blood derived filtered and irradiated platelets from a single male donor due to profound thrombocytopenia and developed TRALI. The aim of this case report is to emphasize the diversity of the possible lethal complications during treatment of MAS in patients with sJIA.

## 2 Case report

Our patient, a 6-year-old girl with a one-year medical history of sJIA diagnosed by the International League of Associations for Rheumatology classification criteria (13) was rehospitalized due to the first relapse of sJIA that developed at 13 months of disease onset. Laboratory evaluation revealed a significant increase in acute phase reactants (erythrocyte sedimentation rate 94 mm/h, C-reactive protein 234 ng/L, procalcitonin 22.96 ng/ml, ferritin 3,272 μg/L), increased D-dimers (4.11 mg/L) and fibrinogen (8.1 g/L), and normal levels of aspartate transaminase, alanine transaminase and lactate dehydrogenase, accompanied by white blood cell and platelet counts, and no anemia. Due to immunosuppressive therapy and systemic inflammatory response, our first aim was to exclude infection; following sampling for blood cultures, polymerase chain reaction for Epstein-Barr virus and cytomegalovirus, empirical treatment was first started with ceftriaxone i.v., later switched first to tazocine, and then to meropenem and vancomycin following consultation with an infectious disease specialist. Early evaluation of antibacterial treatment showed no effect on clinical presentation and inflammatory markers; thus pulse corticosteroid therapy was administered again (30 mg/kg for three days) with only partial effect on the disease presentation and inflammatory markers. Therefore, treatment with an interleukin-1 receptor antagonist (anakinra) was sought from the Committee for Drug Approval, as this drug was not at the time registered for sJIA treatment in our country. Following the Committee approval and signed informed consent by the parents, anakinra was administered at a dose of 100 mg s.c. once daily. This treatment led to normalization of fever and inflammatory markers within 3 days.

**Figure 1:**
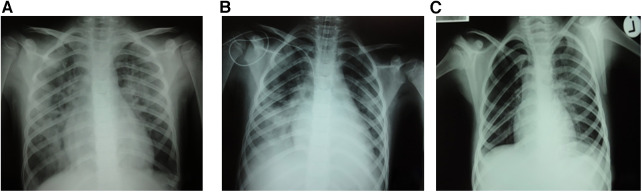
Bilateral pulmonary infiltrates post-transfusion (**A**) and immediately after intubation (**B**) with significant radiological improvement 40 hours post-event (**C**).

**Figure 2:**
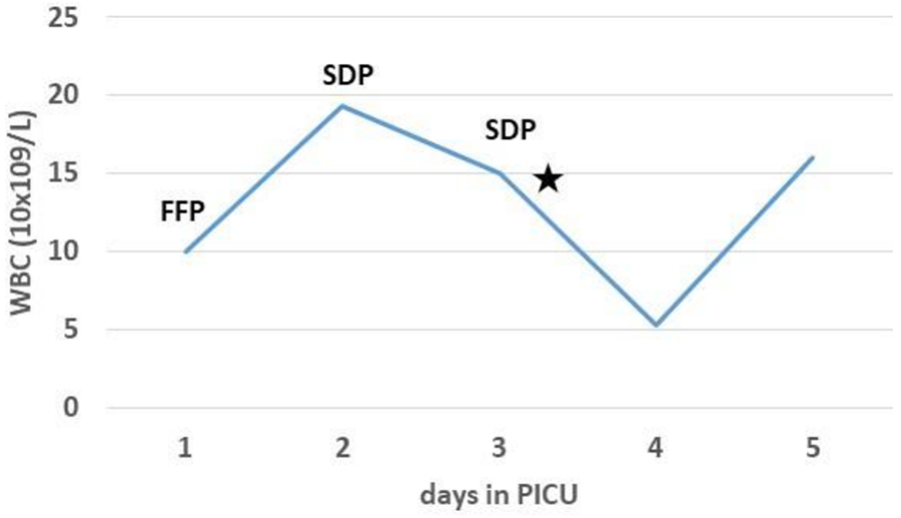
Laboratory investigations showed transitory leukopenia (⋆) following transfusion of single donor platelets (SDP). WBC, white blood cells; FFP, fresh frozen plasma.

Three weeks after the introduction of anakinra, the patient became febrile again with chills, mild hepatosplenomegaly, and significantly increased inflammatory markers (C-reactive protein 493.7 ng/L, procalcitonin 50.32 ng/ml). Additional laboratory evaluation confirmed MAS in our patient during the first relapse of sJIA (Table 1, column “Flare 1”) and she was transferred to the pediatric intensive care unit for further treatment under intensive monitoring. Heart ultrasound revealed pericarditis and hypertrophic myocardiopathy accompanied by significant increase in N-terminal prohormone of brain natriuretic peptide of >35,000 ng/L and normal troponin. On the first day at the intensive care unit, she received fresh frozen plasma for correction of hypofibrinogenemia, and on days 2 and 3 whole blood derived filtered and irradiated platelets stored in a single donor plasma from a male donor due to rapid drop in platelet count (from 178 to 36 × 10^9^ /L within 12 h). Approximately one hour post-infusion of platelets on day 3, she started coughing and rapidly became progressively dyspneic. Physical examination of the patient revealed tachycardia, tachypnea, and diffuse bilateral crepitations throughout her lungs. Hypoxemia was evident with peripheral oxygen saturation persistently below 90% and increasing oxygen requirements. The administration of intravenous furosemide and hydrocortisone had no effect on her symptoms. Urgent chest radiograph (Figure 1A) showed bilateral inhomogeneous opacities but no cardiomegaly, and she was promptly intubated and placed on mechanical ventilation for a total of 30 h (Figure 1B). There was no evidence for acute heart failure secondary to ischemic event or circulatory overload, so the diagnosis of transfusion-associated circulatory overload (TACO) was unlikely. Microbial analysis for possible sepsis was negative. Laboratory evaluation revealed transitory leukopenia (Figure 2). The patient's pulmonary condition rapidly improved and a radiograph taken 40-h post-event showed marked resolution of the airspace shadowing (Figure 1C). She was extubated and transferred to the Immunology and Rheumatology Department one day later, with no further pulmonary sequels. Based on the relevant criteria for TRALI at the time of presumed transfusion complication, blood immunology was performed to detect Human Leukocyte Antigen (HLA) class I, class II, and human neutrophil antigen (HNA) antibodies in the donor's and patient's plasma by using the following tests: granulocyte immunofluorescence test, granulocyte agglutination test, monoclonal immobilization of granulocyte antigen, and enzyme immunoassay kit (Lifecodes Quick-screen and B-screen, Immucor GTI Diagnostics, Wisconsin, USA) (14). Anti-HLA class I, class II and/or HNA antibodies were not detectable either in donor's or patient's plasma, suggesting that in our patient, TRALI may have resulted from a non-immune mechanism in which the recipient had previous and/or existing inflammatory pathological condition favoring the development of type II TRALI (9).

**Table 1:** Clinical manifestations, laboratory values and treatment administered for systemic juvenile idiopathic arthritis (sJIA), in sJIA flare, and following diagnosis of macrophage activation syndrome (MAS).

	Onset	Flare No. 1	Flare No. 2	Flare No 3	Flare No. 4
	Jan 8, 2011	Feb 10, 2012	Sep 13, 2012	Jul 8, 2013	Feb 20, 2015
Clinical manifestations of sJIA					
Fever (duration, days)	16	1	1	1	4
Arthritis (Y/N)	N	N	N	N	N
Arthritis at any time during episode (Y/N)	N	N	N	N	N
Arthralgia at onset (Y/N)	Y	Y	N	N	N
Arthralgia at onset (duration, days)	5	2	N	N	N
Evanescent rash (Y/N)	Y	Y	N	Y	N
Generalized lymphadenopathy at onset (Y/N)	N	N	N	N	N
Hepatomegaly/splenomegaly at onset (Y/N)	Y	N	N	N	N
Serositis at onset (Y/N)	N	N	N	N	N
Sore throat at onset (Y/N)	N	N	N	N	N
Liver dysfunction at onset (Y/N)	N	N	N	N	N
Central nervous system involvement	N	Y	Y	N	N
Infectious trigger (Y/N)	Y	N	N	N	Y
Infectious agent	Y^1^	N	N	N	Y^2^
Laboratory tests					
ANA positivity (Y/N)	N	N	N.P.	N.P.	N.P.
Ferritin (ng/ml)	2,493	3,272	72.3	585	171.8
LDH (UI/L)	256	276	237	224	189
AST (UI/L)	22	27	61	32	15
GGT (UI/L)	14	18	29	16	9
Triglycerides (mmol/L)	1.15	1.02	1.3	0.38	1.2
WBC (×10^9^/L)	14.28	12.8	7.5	10.8	9.2
Neutrophils (×10^9^/L)	9.85	11.36	4.8	8.06	5.87
Platelets (×10^9^/L)	380	381	315	340	332
Hemoglobin (g/L)	109	113	96	124	101
CRP (mg/L)	151.5	234	44.6	42.2	95.4
Erythrocyte sedimentation rate (mm/h)	35	94	29	20	32
Fibrinogen (g/L)	6.2	8.1	4.2	4.3	5.6
ALT (UI/L)	11	17	39	32	9
D-dimer (mg/L)	3.8	4.11	2.5	2.02	0.27
MAS (Y/N)	Y	Y	Y	Y	N
Laboratory tests at diagnosis of MAS					
Ferritin (ng/ml)	3,686	3,382	730	5,300	
LDH (UI/L)	677	485	502	688	
AST (UI/L)	166	74	261	338	
GGT (UI/L)	79	100	102	45	
Triglycerides (mmol/L)	1.01	3.04	2.06	1.5	
WBC (×10^9^/L)	6.74	10	2.9	6.4	
Neutrophils (×10^9^/L)	4.78	6.1	2	5.46	
Platelets (×10^9^/L)	101	178	113	92	
Hemoglobin (g/L)	90	100	98	109	
CRP (mg/L)	141	342	213.9	207.6	
Erythrocyte sedimentation rate (mm/h)	19	12	48	15	
Fibrinogen (g/L)	1.14	1.9	1.7	1.4	
ALT (UI/L)	69	119	108	117	
D-dimer (mg/L)	4.44	7.83	9	9	
Treatment of sJIA Y/N					
Nonsteroidal anti-inflammatory drugs	Y	N	N	N	N
IV methylprednisolone	Y	Y	Y	Y	Y
Oral prednisone	Y	Y	Y	Y	Y
Tocilizumab	N	N	N	N	N
Anakinra	N	N	N	N	N
Cyclosporine A	N	N	N	N	Y
Treatment of MAS Y/N					
Nonsteroidal anti-inflammatory drugs	N	N	N	N	
IV methylprednisolone (Y/N)	Y	N	Y	Y	
Oral prednisone (Y/N)	Y	Y	Y	Y	
Tocilizumab	N	N	N	N	
Anakinra	N	Y	N	N	
Cyclosporine A	Y	Y	Y	Y	

Y, yes; N, no; ANA, antinuclear antigen; LDH, lactate dehydrogenase; AST, aspartate aminotransferase; GGT, gamma-glutamyl transferase; WBC, white blood cells; CRP, C-reactive protein; ALT, alanine aminotransferase.

In this patient, TRALI developed during the first flare of sJIA complicated by MAS.

Following clinical suspicion of TRALI in our patient, the Croatian Hemovigilance Network (CHN) members immediately contacted all medical centers in our country to see if any other patient having received blood components from the same male donor had TRALI, but no such complication was reported. Treatment of MAS was continued with repeated pulse dose of steroids for three days, followed by prednisolone p.o. and cyclosporine A, and this approach led to successful control of sJIA and MAS as its complication within 10 days. According to the recommendation obtained from Transfusion Service, physicians were instructed to pay due attention to the patient's risk factors for acute lung injury and implement evidence-based national transfusion practices avoiding transfusions when they were not vitally indicated (15).

During 12-year follow-up, our patient had a total of four relapses of sJIA (Table 1). Following the first relapse complicated with MAS and suspected type II TRALI, additional two flares of sJIA with MAS were promptly recognized and treated. The patient's parents refused therapy with anakinra, so treatment with steroids and cyclosporine A was administered during these flares. Our patient had no need of transfusion of blood derivatives during the follow-up. Based on the severe and relapsing presentation of the disease complicated by MAS, which could be considered as refractory sJIA (16), additional evaluation for familial hemophagocytic lymphohistiocytosis was performed at Karolinska University Hospital, Huddinge, Sweden, but the NK cell count, NK cell-mediated cytotoxicity, degranulation of NK- and T cells, perforin and SLAM-associated protein were all normal. The patient has been symptom-free and without any therapy for the last eight years.

## 3 Discussion

Transfusion-related acute lung injury is the leading cause of transfusion-associated mortality (10). TRALI was initially defined based on clinical and radiological parameters at the 2004 Consensus Conference organized by the National Heart, Lung and Blood Institute Working Group on TRALI, as a newly developed acute lung injury (ALI)/acute respiratory distress syndrome (ARDS) within six hours of blood product transfusion (8). Most cases of TRALI have been reported in adult patients, but rising awareness of TRALI in children, especially in the setting of hematologic diseases, has led to its recognition in children as well (17, 18).

Various mechanisms have been proposed for the pathogenesis of TRALI. The priming step consists of previous and/or existing inflammatory pathological conditions or external factors attracting leukocytes to lung vessels and creating conditions favorable for the second step, in which anti-HLA, anti-HNA antibodies, various biologically active mediators secreted by cells or present in transfused blood products lead to the stress of leukocytes and consequent inflammation of lung epithelia and pulmonary edema. In addition, monocyte and macrophage activation has also been implicated in the pathophysiology of TRALI. Several activation mechanisms of these cells have been described, such as those mediated by pathogen-associated and damage-associated molecular patterns and their receptors, antibodies, complement, as well as neutrophil extracellular traps (10, 19–21).

The most common pulmonary complications in sJIA patients include pleural effusion and pleuritis. More recently, pulmonary artery hypertension, interstitial lung disease and alveolar proteinosis have been reported in these patients with increased frequency and being associated with mortality (22, 23). However, our patient had no signs and symptoms of respiratory disease prior to MAS onset, which may have contributed to ALI development or mimic TRALI.

Based on a recent significant progress in defining the factors contributing to TRALI pathophysiology, patients with sJIA complicated by MAS have many risk factors for TRALI onset since these conditions share many overlapping mechanisms. An immune feature of MAS in patients with sJIA is excessive activation and proliferation of macrophages and T lymphocytes. Massive hypercytokinemia is strongly associated with MAS pathogenesis, particularly the overproduction of cytokines such as interleukin (IL)-1, IL-6, IL-18, interferon-γ, and tumor necrosis factor-α. Inadequate production of IL-10, a regulatory cytokine to counter-regulate interferon-*γ*, might be related to the development of MAS (24). Several of these cytokines (IL-6, IL-10) are also important in the pathophysiology of TRALI (21). Based on the several overlapping mechanisms involved in the pathophysiology of MAS in sJIA and those identified as triggers/risks for TRALI, our patient had significant recipient-related risk factors that could have contributed to the onset of TRALI.

The management of TRALI is mainly supportive by using oxygen and ventilatory support, and most cases show improvement within the first few hours and completely resolve within 1–4 days (10). Our patient improved markedly after 40 h of oxygen therapy. Plasma-containing components from female donors with leukocyte antibodies were responsible for the majority of immune (antibody mediated) TRALI fatalities before mitigation strategies had been implemented. Since 2006, the CHN has introduced mitigation approach for TRALI in our country following the international recommendations. Serology investigation for anti-leukocyte antibodies in suspected TRALI cases has been implemented in the Croatian Institute of Transfusion Medicine since 2008, according to the International Society of Blood Transfusion Granulocyte Working Party recommendation (14). As already described, our patient received platelets stored in a single donor plasma. Starting from 2015, transfusion with platelets suspended in platelet additive solution has been introduced in our country, as this additional strategy can reduce TRALI-associated morbidity (25).

Based on the clinical presentation of new ARDS within six hours of platelet transfusion in our patient, we were able to initiate necessary investigations and identify the possible implicated mechanism of TRALI. This analysis revealed no presence of anti-HNA or anti-HLA class I and class II antibodies in either donor's or patient's plasma, suggesting that in our patient, TRALI may have resulted from a non-immune mechanism.

TRALI has been recently described in an adult patient with systemic lupus erythematosus complicated by autoimmune hemolytic anemia following infusion of packed red blood cells and intravenous immunoglobulins (26). This report further emphasizes the possible common pathophysiological pathway of TRALI with autoimmune and/or autoinflammatory diseases.

There are currently no specific international transfusion recommendations for children with sJIA complicated by MAS. A recently published EULAR/American College of Rheumatology report provides the points to consider as guidance for supportive therapy based on the available data and expert opinion, including blood product transfusion, and suggests implementation of national recommendations and guidelines (27).

In conclusion, our findings underline the importance of clinical diagnosis of TRALI in a life-threatening condition, i.e., sJIA complicated by MAS, in case of a new-onset ALI during the treatment of MAS with blood derivatives. Further research is necessary to determine whether specific transfusion threshold guidelines in these critically ill children are required. Until these data are available, the critical and restrictive approach for transfusion of blood products according to the local/national recommendations should be followed.

## Data Availability

The original contributions presented in the study are included in the article/Supplementary Material, further inquiries can be directed to the corresponding author.
